# Retroperitoneal abscess with concomitant hepatic portal venous gas and rectal perforation: a rare triad of complications of acute appendicitis. A case report

**DOI:** 10.1186/1749-7922-5-3

**Published:** 2010-01-28

**Authors:** Michele Diana, Alexandre Paroz, Nicolas Demartines, Markus Schäfer

**Affiliations:** 1 Department of Visceral Surgery, Centre Hospitalier Universitaire Vaudois, University Hospital, Lausanne, Switzerland

## Abstract

**Background:**

While retroperitoneal abscess is a known complication, hepatic portal venous gas and rectal perforation have not been reported as a concomitant sequelae of acute appendicitis. Here we report a case of a patient with a perforated appendicitis that was associated with these triad of complications.

**Materials and Methods:**

In addition to report our case, we carefully reviewed the literature in order to detect similar cases and the causes of such rare conditions.

**Results:**

Only 26 cases (including our patient) of acute appendicitis complicated by retroperitoneal abscesses have been published in the English literature between 1955 and 2008. There was one case having hepatic portal venous gas, and one further case with a rectal perforation associated with acute appendicitis. All patients with retroperitoneal abscess presented with non specific clinic symptoms that not revealed any suspicion for such a complicated disease. Hence, delayed diagnosis and treatment are not uncommon.

**Conclusions:**

So far, no patient has been described with such a triad of rare complications related to acute appendicitis. We want to emphasize the insidious onset of retroperitoneal abscess formation, and the need of prompt recognition and adequate treatment to avoid deleterious outcome.

## Introduction

Acute appendicitis is a very common disease with low morbidity and mortality rates in most countries. While uncomplicated appendicitis can easily be treated, complicated appendicitis with perforation and abscess formation remains a challenging treatment. In particular, large abscess and advanced peritonitis often require repeated surgical interventions combined with percutaneous drainage performed by interventional radiology, as well as intensive care and antibiotic treatment. Such treatment is associated with markedly increased complications, e.g. sepsis, prolonged ileus, and adhesion formation [1]. The development of incisional hernia, recurrent bowel obstruction, and impaired fertility rates in female patients are the main adverse events during long-term course [2]. In contrast, bowel necrosis with portal venous gas formation is very rare after complicated appendicitis [3]. Here we report a case of an extensive retroperitoneal abscess formation with rectal perforation and portal venous gas embolization after necrotizing acute appendicitis in a young male patient.

## Case report

A 43-year old man was admitted to the Emergency Department with progressive abdominal pain, nausea, reduction in defecatory frequency and change in stool appearance as hard separate lumps that started almost three weeks before, and in addition, new onset of anal bleeding. There were no preexisting co-morbidities. The patient had tachycardia (up to 140 bpm), arterial hypertension (170/70 mmHg) and fever (38°C). Clinical examination revealed an abdominal distension with a palpable mass in the lower abdomen, as well as signs of peritoneal irritation. The rectal examination was very painful, and an ulcerative lesion was perceived on the anterior rectal wall. Anal bleeding could be confirmed.

The laboratory findings revealed increased C-reactive protein (CRP) levels up to 100 mg/l, leucocytes 8.8 G/l, and serum lactate levels of 4.5 mmol/l.

**Figure 1:**
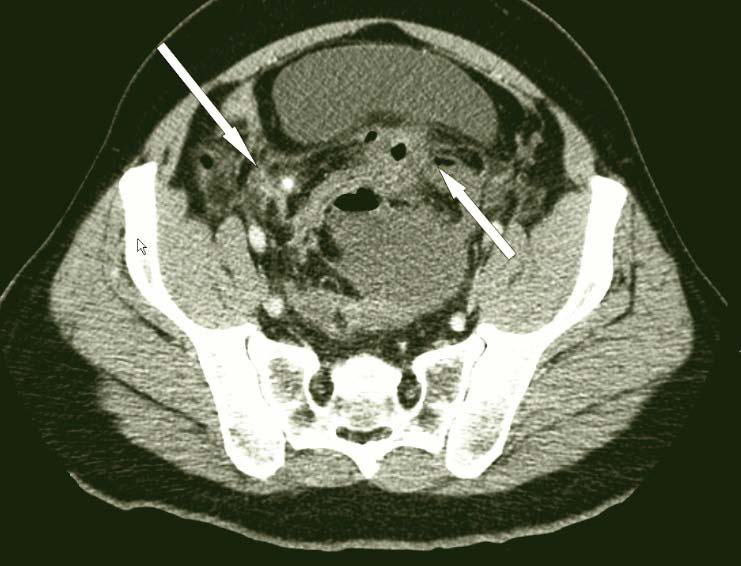
**CT Scan showing a necrotic appendix with a stercolith (long arrow) and anterior wall perforation (short arrow)**.

**Figure 2:**
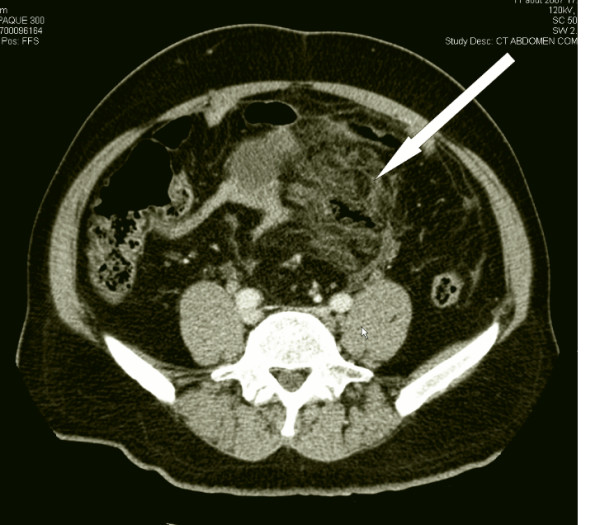
**Retroperitoneal phlegmon with some air bubbles**.

**Figure 3:**
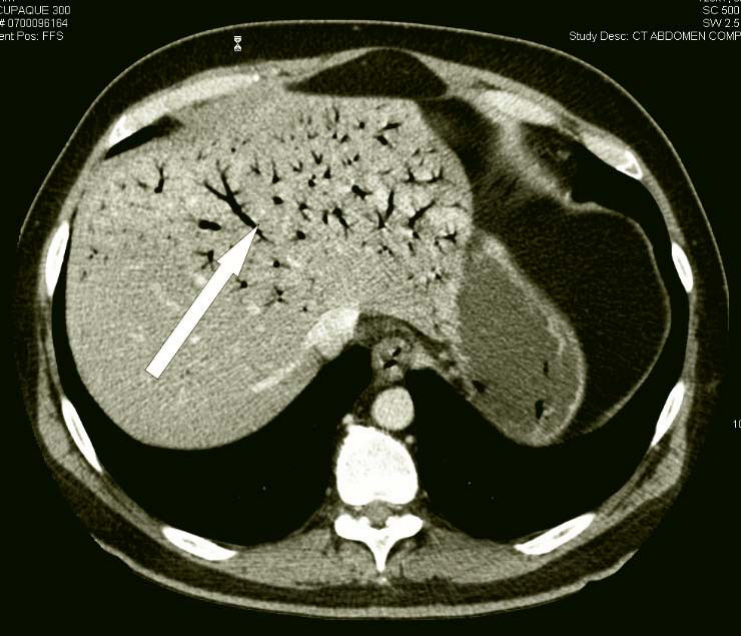
**Hepatic portal venous gas in several intrahepatic portal branches**.

The abdominal CT scan with only IV contrast showed a perforation of the anterior rectal wall, 10 cm proximally from the anorectal border with multiple, partially confluent large abscesses located extra- and retroperitoneally (Figure 1). A significant air collection ascended from the lower pelvis through the retroperitoneal space up to the left kidney (Figure 2). Finally, massive hepatic portal venous gas was detected (Figure 3). Due to a coprolith and local abscess formation, appendiceal perforation was also highly suspected (Figure 1).

The patient underwent emergency laparotomy. Intraoperatively, a necrotizing appendicitis was found with multiple abscess formation in the retroperitoneal space. The abscess extended from the perirectal area in the pelvis up to the left kidney. The sigmoid colon, the upper and mid rectum were surrounded by the abscess. Perforation of the anterior rectal could be confirmed. Sigmoid and the upper two third of the rectum were resected, and a Hartmann's situation created. The appendix was excised and all abscess were drained by widely opening the retroperitoneal space.

Due to the severe sepsis, the patient stayed for three days in the ICU, and another 18 days on the normal ward. Initial blood cultures were positive to Bacterioides fragilis and turned sterile after a week. Cultures of the abscesses were positive to Bacterioides fragilis, Escherichia coli and Streptococcus anginosus. IV antibiotic treatment (Piperacillin-Tazobactam 4.5 g three times per day) was performed for 15 days and a further per os antibiotic treatment (Levofloxacin 500 mg twice per day) was introduced for 7 days. The patient fully recovered, and was finally discharged after 21 days. Restoration of the bowel continuity was performed after 3 months. During follow-up of one year, the long-term course was uneventful.

Histopathology showed a perforated appendicitis with severe peritonitis, as well as large necrosis formation of sigmoid mesenteric adipose tissue and a necrotic ulcer measuring 1 cm square on the anterior wall of the rectum. Since no diverticular disease could be detected, it was strongly assumed that necrotizing appendicitis being the trigger of this massive inflammatory process that also facilitated rectal wall necrosis and stercoral perforation, respectively.

## Discussion and review of the literature

### Retroperitoneal abscess and acute appendicitis

Large retroperitoneal abscess represents a potentially life-threatening complication of hollow viscus organ perforation, e.g. appendicitis [4,5], diverticulitis [6], as well as inflammatory diseases of the pancreas [7] and kidneys [8]. Often its starts as a retroperitoneal phlegmon with few clinical symptoms, hence its timely diagnosis may not be always achieved. Once abscess formation has started, it may spread from the pelvis along the spine and psoas muscle up to the diaphragm and laterally to the abdominal wall since there are no anatomical barriers limiting its penetration. Perforation of the appendix into the retroperitoneal space probably represents one of the commonest reason for large retroperitoneal abscess formation but there are only few reported series in the literature [4]. While its real incidence remains unknown, several risk factors have been identified to promote large abscess formation, such as diabetes, alcohol abuse, liver cirrhosis, malignancy, chronic renal failure, and immunosuppressive therapy [9].

Hsieh et al. recently reported two cases and summarized the literature, whereby they found only additional 22 cases [4]. The main clinical features are the delayed diagnosis (mean time until diagnosis of 16 days), symptoms are dependent on the localization of the abscess and often unspecific, extension of abscess formation into the thigh and perinephritic space, and an increased disease-related mortality of 19%. Similar to our case, final diagnosis of retroperitoneal perforation originating from acute perforated appendicitis is often only achieved during surgical exploration. However, it remains unclear, who an otherwise healthy young patient can develop such a major abscess without having more clinical symptoms.

### Hepatic portal venous gas and acute appendicitis

The presence of air bubbles in the extrahepatic and/or intrahepatic portal venous system is primarily a radiological finding that is detected by performing an abdominal CT scan for various reasons. Despite portal venous gas is generally a late feature of advanced intestinal necrosis with an increased mortality, there are various other clinical conditions that may also cause portal venous gas formation, i.e. inflammatory bowel disease, biliary tract infections, cardiac and liver transplantation, acute pancreatitis, and blunt abdominal trauma [10]. It is assumed that gas may enter the portal venous system by an intestinal mucosal damage and increased intraluminal pressure, or gas-forming bacteria may translocate through the bowel wall during abdominal sepsis. While bowel necrosis was the predominant reason for portal venous gas formation, non-ischemic reasons have become more frequent during recent decades [11]. Due to the latter reasons, overall morbidity decreased from 75% to 39%.

Portal venous gas formation due to perforated appendicitis has been previously reported in two cases [3,12]. In our patient, portal venous gas formation could potentially be induced by both, perforated appendicitis and rectal perforation, respectively. However, it was assumed that rectal perforation was a secondary complication of the retroperitoneal abscess which occurred as a sequelae of perforated appendicitis.

### Rectal perforation and acute appendicitis

Rectal perforation and necrosis represents an extremely rare event after retroperitoneal abscess formation. So far, only one case of rectal necrosis and simultaneous pelvic abscess as a consequence of perforated appendicitis was published in 1968 by Gostev [13]. In our patient, it remains somewhat unclear, which was the pathophysiology of rectal perforation. Ischemia, pre-existing inflammatory bowel disease, and manipulation as the commonest reasons could be excluded. Thus, impacted stool due to abscess-related impaired bowel motility caused a so-called stercoral perforation.

## Conclusion

In conclusion, this patient presented with three very rare complications of acute appendicitis that all occurred at the same time. Despite the delayed diagnosis, the final outcome was good due to the rapid surgical intervention that aimed to control all infectious areas in order to assure patient's survival.

## Competing interests

The authors declare that they have no competing interests.

## Authors' contributions

MD and AP drafted the manuscript, ND et MS critically revised the manuscript. All authors read and approved the final manuscript.
